# Association between alkaline phosphatase/albumin ratio and the prognosis in patients with chronic kidney disease stages 1–4: results from a C-STRIDE prospective cohort study

**DOI:** 10.3389/fmed.2023.1215318

**Published:** 2023-09-20

**Authors:** Xue Xue, Jia-Xuan Li, Jin-Wei Wang, La-Mei Lin, Hong Cheng, Dan-Fang Deng, Wen-Cheng Xu, Yu Zhao, Xin-Rong Zou, Jun Yuan, Lu-Xia Zhang, Ming-Hui Zhao, Xiao-Qin Wang

**Affiliations:** ^1^The First Clinical Medical School, Hubei University of Chinese Medicine, Wuhan, China; ^2^School of Clinical Traditional Chinese Medicine, Hubei University of Chinese Medicine, Wuhan, China; ^3^Renal Division, Department of Medicine, Peking University First Hospital, Beijing, China; ^4^Institute of Nephrology, Peking University, Beijing, China; ^5^Key Laboratory of Renal Disease, National Health Commission of China, Beijing, China; ^6^Key Laboratory of Chronic Kidney Disease Prevention and Treatment, Peking University, Ministry of Education, Beijing, China; ^7^Research Units of Diagnosis and Treatment of Immune-Mediated Kidney Diseases, Chinese Academy of Medical Sciences, Beijing, China; ^8^Department of Nephrology, Affiliated Hospital of Hubei University of Chinese Medicine, Hubei Provincial Hospital of Traditional Chinese Medicine, Wuhan, China; ^9^Department of Nephrology, Renmin Hospital of Wuhan University, Wuhan University, Wuhan, China; ^10^National Institute of Health Data Science at Peking University, Beijing, China; ^11^Hubei Key Laboratory of Theory and Application Research of Liver and Kidney in Traditional Chinese Medicine, Hubei Provincial Hospital of Traditional Chinese Medicine, Wuhan, China

**Keywords:** alkaline phosphatase-to-albumin ratio, chronic kidney disease, end-stage kidney disease, major adverse cardiovascular and cerebrovascular events, all-cause death, cohort study

## Abstract

**Background:**

The alkaline phosphatase-to-albumin ratio (APAR) has been demonstrated to be a promising non-invasive biomarker for predicting prognosis in certain diseases. However, the relationship between APAR and prognosis in non-dialysis chronic kidney disease (CKD) patients remains unclear. This study aims to identify the association between APAR and prognosis among CKD stages 1–4 in China.

**Methods:**

Patients with CKD stages 1–4 were consecutively recruited from 39 clinical centers in China from 2011 to 2016. New occurrences of end-stage kidney disease (ESKD), major adverse cardiovascular and cerebrovascular events, and all-cause deaths were the outcome events of this study. Subdistribution hazard competing risk and Cox proportional hazards regression models were adopted.

**Results:**

A total of 2,180 participants with baseline APAR values were included in the analysis. In the primary adjusted analyses, higher APAR level [per 1-standard deviation (SD) increase in natural logarithm transformed (ln-transformed) APAR] was associated with 33.5% higher risk for all-cause deaths [adjusted hazard ratio (HR) 1.335, 95% confidence interval (CI) 1.068–1.670]. In addition, there was evidence for effect modification of the association between APAR and ESKD by baseline estimated glomerular filtration rate (eGFR) (*P* interaction < 0.001). A higher APAR level (per 1-SD increase in ln-transformed APAR) was associated with a greater risk of ESKD among participants with eGFR ≥ 60 ml/min/1.73 m^2^ (adjusted SHR 1.880, 95% CI 1.260–2.810) but not in eGFR < 60 ml/min/1.73 m^2^.

**Conclusion:**

Higher APAR levels in patients with CKD stages 1–4 seemed to be associated with an increased risk of all-cause death. Thus, APAR appears to be used in risk assessment for all-cause death among patients with CKD stages 1–4.

## 1. Introduction

Chronic kidney disease (CKD) has become a major public health problem worldwide, with a prevalence rate of over 10% among the general population in developed countries and 8.2% in China (1, 2). With the increasing prevalence of diabetes, hypertension, obesity, and an aging population, the prevalence of CKD is projected to grow continuously in the near future (3). Regardless of the underlying etiology, CKD is slowly progressive and leads to irreversible nephron loss, end-stage kidney disease (ESKD), and/or death (3). When CKD progresses to stage 5, therapies of replacement are required, such as transplantation or dialysis. Cardiovascular disease (CVD) is the most common and fatal complication of patients with CKD (4). CVD risk is markedly increased even at the early stages of CKD, and CVD deaths account for half of all known causes of mortality in ESKD (4). Therefore, it is of great value to explore the factors affecting the prognosis of patients with CKD for the prevention and treatment of CKD.

Alkaline phosphatase (ALP) is a routine biochemical examination indicator that can be secreted by normal tissues, including the liver, small intestine, kidney, and bone. ALP levels are elevated when these tissues are affected by trauma, inflammation, metabolic disorders, or malignancy (5). Circulating ALP is a robust risk marker for cardiovascular disease and all-cause mortality in the CKD population (6). Albumin (ALB), another important biochemical indicator, has been widely recognized as an evaluation marker for nutritional and inflammatory status (7, 8). With systemic inflammation, ALB eliminates nitrogen species and active oxygen, while it reduces itself (9, 10). The alkaline phosphatase-to-albumin ratio (APAR) is a novel and easily available inflammation-based score. It can reflect the systemic inflammatory response and nutritional status of the patient (11). Many studies have shown that APAR is a promising non-invasive biomarker for predicting cancer prognosis (11–13). In 2017, researchers analyzed 354 patients with pancreatic ductal adenocarcinoma undergoing curative resection through a retrospective cohort study. The results indicated that patients with higher serum APAR levels would probably sustain poor overall survival (11).

Recently, researchers have found that APAR is also used to predict adverse outcomes of coronary artery disease (14, 15). The result from a prospective cohort study of 2,162 patients from China found that acute coronary syndrome patients with higher APAR values had higher all-cause mortality and cardiac mortality (14). It is well-known that CVD is closely related to CKD (4). Inflammation, vascular calcification, endothelial dysfunction, and poor nutritional status are all closely linked with the occurrence of cardiovascular events in patients with CKD (16). Abnormal expression of alkaline phosphatase and albumin is often linked with the abovementioned pathological states in renal and cardiovascular diseases (5, 7–10, 17). We, therefore, hypothesized that APAR might be associated with the prognosis of patients with CKD.

To date, APAR levels and the risk of prognosis in non-dialysis CKD patients have not been identified. Therefore, we aimed to investigate the association between APAR levels and the prognosis in patients with CKD stages 1–4 in China, using the Chinese cohort study of CKD (C-STRIDE), where ESKD, major adverse cardiovascular and cerebral events (MACCEs), and death were well-recorded. We hypothesized that there may be an independent correlation between elevated APAR levels and outcome events (new occurrences of ESKD, MACCEs, and death) in patients with CKD.

## 2. Methods

### 2.1. Study design and population

The design and methods of the C-STRIDE study were published in detail in a previous study (18). C-STRIDE is a multicenter prospective cohort study initiated in November 2011 that includes patients with stages 1–4 CKD and various etiologies in 39 clinical centers in 22 provinces around China. This study was approved by the ethics committee of Peking University First Hospital.

Before enrolment, all participants were informed of the purpose of the study and signed informed consent forms. The content of the cohort study was reported in light of the Strengthening the Reporting of Observational Studies in Epidemiology (STROBE) (19).

Participants must meet the following criteria to be eligible for enrollment: 18–74 years old and have a specified estimated glomerular filtration rate (eGFR) range based on different CKD etiologies. For patients with diabetic kidney disease (DKD) (20), the defining eligibility is 15 ml/min/1.73 m^2^ ≤ eGFR < 60 ml/min/1.73 m^2^ or eGFR ≥ 60 ml/min/1.73 m^2^ with “nephrotic range” proteinuria, defined as 24 h urinary total protein ≥ 3.5 g or urinary albumin creatinine ratio (UACR) ≥ 2,000 mg/g. For patients with glomerulonephritis (GN) (21), the eGFR should be ≥15 ml/min/1.73 m^2^. For non-GN and non-DKD patients, 15 ml/min/1.73 m^2^ ≤ eGFR < 60 ml/min/1.73 m^2^ was the cutoff for enrollment. Stages of CKD were determined by the Kidney Disease Improving Global Outcomes (KDIGO) classification (22). Participants were excluded if one of the following conditions were met: New York Heart Association (NYHA) Class III or IV heart failure; CKD caused by systemic inflammatory illness or autoimmune disease; patient treated with immunosuppressive agents in the preceding 6 months to treat renal or immune disease; self-reported or known diagnosis of human immunodeficiency virus (HIV) infection and/or acquired immune deficiency syndrome (AIDS); isolated hematuria; self-reported or known diagnosis of cirrhosis; pregnant or lactating women; malignancy treated with chemotherapy within the last 2 years; renal or other transplantation; hereditary kidney disease; and participation in an intervention clinical trial.

### 2.2. Data collection

All C-STRIDE study data collection was completed by trained research staff according to the study protocol (18). Questionnaire surveys, anthropometric measurements, and laboratory parameters for each subject were recorded during the study visit. Blood specimens, spot urine, and 24 h urine samples were collected locally at each subcenter and then transported by cold chain to the central laboratory of Peking University First Hospital. Measurements of all urine and serum biomarkers were tested centrally at Peking University First Hospital. APAR was calculated with the following equation: APAR = serum ALP level/serum ALB level ratio. The spot urine albumin/creatinine ratio (UACR) was calculated. eGFR was determined with the CKD-EPI (Chronic Kidney Disease Epidemiology Collaboration) equation using serum creatinine (Scr) measured by Roche enzymatic method (23).

### 2.3. Definition of covariates

Cumulative smoking of more than 100 cigarettes was defined as “tobacco use”. Body mass index was weight (kg) divided by height (m) squared. “Diabetes” was defined as a fasting blood glucose level ≥ 7.0 mmol/L at the baseline visit, a history of diabetes, or taking antidiabetic drugs or insulin for the past 2 weeks. “History of cardiovascular disease” included previous diagnoses of myocardial infarction, congestive heart failure, arrhythmia, cerebrovascular disease, and/or peripheral arterial disease. The abovementioned information was the self-reported results of the interviewees. The classification for the eGFR was determined according to the Kidney Disease Improving Global Outcomes guideline (22). Stages of CKD were divided as follows: ≥90 (stage 1), 60–<90 (stage 2), 45–<60 (stage 3a), 30–<45 (stage 3b), and 15–<30 (stage 4), according to the eGFR levels (ml/min/1.73 m^2^).

### 2.4. Outcome variables

The outcome events of the study were new onset ESKD, MACCEs, and all-cause death. ESKD was a composite renal end-point comprising initiation of renal hemodialysis [the International Classification of Disease codes (ICD) Z49.1], peritoneal dialysis (ICD Z49.1), or transplantation (ICD Z94.0). In summary, the initiation of renal replacement therapies (RRT) was one of the end-point outcomes in the current study.

MACCEs include non-fatal acute myocardial infarction (ICD I21), unstable angina (ICD I20), arrhythmia (ICD I44–I49) (resuscitated cardiac arrest, ventricular fibrillation, sustained ventricular tachycardia, paroxysmal ventricular tachycardia, an initial episode of atrial fibrillation or flutter, severe bradycardia, or heart block), hospitalization for congestive heart failure (ICD I50), cerebrovascular events (ICD I60–I69) (intraparenchymal hemorrhage, subarachnoid hemorrhage, and cerebral infarction), and peripheral vascular diseases (ICD I73).

Researchers followed up with the participants every 6 months to investigate the occurrence of outcome events. The follow-up method was telephone inquiry or when the patients came to the clinic for reexamination. If the patient could not be contacted for more than 6 months, they were defined as “lost to follow-up”, with the date of the last follow-up used for censoring. When there was an outcome event reported, it was necessary to collect and record relevant medical records and examination results, such as surgical records, hospitalization records, dialysis orders, and death certificates. Reported clinical events were adjudicated by the independent committee of specialist physicians in the coordinating center at Peking University First Hospital. If several MACCEs or different modes of RRT occurred, the first event was applied as the index event. The outcomes were followed up until 31 December 2017, to guarantee at least 1 year of follow-up for the participants, censoring for all the outcomes.

### 2.5. Statistical analyses

The absolute value of either skewness or kurtosis ≥ 3 was used to ascertain whether continuous variables were normally distributed. Continuous variables in normal distribution were reported as mean ± standard deviation, variables in skewed distribution by median (Quartiles 1 and 3), and categorical variables by frequency (percentage). The median of APAR was used as a cutoff for binary grouping. Baseline characteristics of patients with CKD were summarized according to the binary grouping of baseline APAR levels. Independent sample *t*-tests were applied to compare the two groups of continuous variables in the normal distribution. Mann–Whitney *U*-tests were used to compare continuous variables in a skewed distribution. Chi-square tests were applied for categorical data. The incidences of three end-point events were expressed as the number of events per 100 person-years, and the log-rank test was used to compare the incidences by APAR levels. APAR was analyzed not only as a continuous variable but also as a categorical variable to explore its association with prognosis in patients with CKD stages 1–4. The time of follow-up in this study started from the baseline and ended at the occurrence of either of the three end-point events, loss of follow-up, or on 31 December 2017.

The subdistribution hazard competing risk regression models were used to determine the association of natural logarithm-transformed APAR levels (or APAR categories) with two outcomes of ESKD and MACCEs, considering death as a competing event. In addition, we used a standard Cox proportional hazards regression model to analyze the outcome of all-cause deaths. Before entering regression models, missing data were filled with the mean or median for continuous variables or with a separate category for categorical variables, and values of variables with skewed distribution were natural logarithm transformed (ln-transformed). The strength of risk associations was reported as subhazard ratios (SHRs) and hazard ratios (HRs) with 95% confidence intervals (CIs). Unadjusted and adjusted hazard models were fit to discuss the relationship between APAR levels and end-point events.

Adjusted model 1 included age (continuous) and sex (male vs. female). Adjusted model 2 included age, sex, body mass index (continuous), diabetes (yes vs. no), hypertension (yes vs. no), cardiovascular disease (CVD) history (yes vs. no), tobacco use (yes vs. no), renin-angiotensin-aldosterone system inhibitor use (yes vs. no), ln-transformed low-density lipoprotein cholesterol (continuous), CKD etiologies (diabetic kidney disease vs. primary glomerulonephritis vs. others), ln-transformed albumin-to-creatinine ratio (continuous), and hemoglobin (continuous). Adjusted model 3 included variables in model 2 plus the baseline eGFR (continuous). Adjusted model 3 was the primary model. Fine-Gray and log-rank tests were used to compare the association of APAR with end-point events.

We hypothesized that the effect of APAR may be modified by baseline age, sex, eGFR, CVD, or etiology. We intended to test for the effect modification of the association between APAR levels and end-point events according to baseline age, sex, eGFR, CVD, or etiology through the inclusion of cross-product terms in the primary model. If there were effect modification factors (all interaction terms *P* < 0.05), further subgroup analysis should be conducted.

Additionally, sensitivity analysis was performed to validate our main finding. An exploratory model was fitted with further adjustments for variables associated with CKD prognosis. The variable of ln-transformed neutrophil-to-lymphocyte ratio (continuous) was added to adjusted model 3 to determine changes in the effect estimate for APAR with outcomes.

In this study, proportionality of hazards (PH) was assessed for each variable in all models. Schoenfeld residuals and log-log plots were visually inspected for potential time-variant biases. The cox.zph function was used to test the PH assumption for covariates and the whole regression models. For covariates that violated the PH assumption, the corresponding time interaction terms were included in the models. Moreover, restricted cubic spline (RCS) was applied to test the relationship between ln-transformed APAR levels and end-point events based on Cox proportional hazards models. A non-linear relationship was considered if the *P*-value for the non-linear value was <0.05. All data analyses were performed using R software version 4.2.2. A two-sided *P*-value of <0.05 was considered statistically significant.

## 3. Results

### 3.1. Screening and enrollment

**Figure 1:**
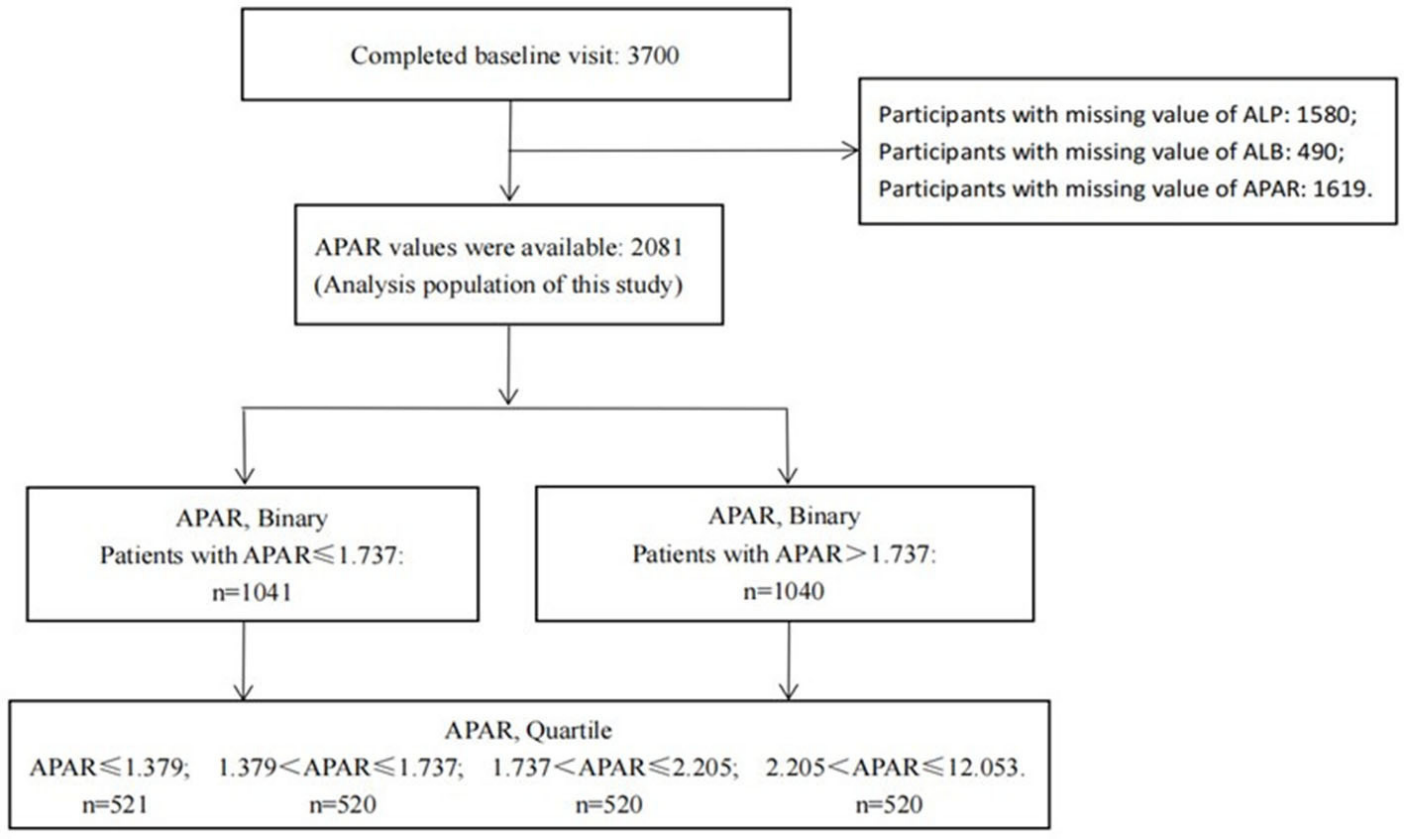
Flowchart of participant selection. APAR, alkaline phosphatase-to-albumin ratio; ALP, alkaline phosphatase; ALB, albumin.

In total, 3,700 patients with CKD stages 1–4 were enrolled from 39 clinical centers in China between 2011 and 2016, including 2,081 patients with baseline APAR measurements and 1,619 patients with missing baseline APAR values. New occurrences of ESKD, MACCEs, and all-cause death were well-recorded by the end of 2017 in this study. A total of 2,081 participants with APAR data were used as the analysis population for this study. A flow chart of participant selection is shown in Figure 1. Baseline characteristics of participants with and without APAR data were compared. Characteristics of age, albumin-to-creatinine ratio (ACR), and low-density lipoprotein cholesterol (LDL-C) were significantly different between them (Supplementary Table 1). In detail, patients with missing APAR data (*n* = 1619) were slightly younger, with lower ACR and LDL-C levels than those with APAR data (all *P*-values < 0.05).

### 3.2. Baseline characteristics

In total, 2,081 participants were included in this analysis, with a mean age of 50.36 ± 14.13 years and male predominance (58.29%). The main etiology was primary glomerulonephritis (52.43%), and the majority of the patients were in CKD stages 3b−4 (53.72%). Baseline characteristics of the demographics, clinical risk factors, medication, and laboratory detection indicators among the total population and population stratified by APAR levels (binary) are presented in Table 1. Participants with higher baseline APAR levels (top binary) were more likely to be older, of male sex, have a history of diabetes and hypertension, and tend to have higher Scr, ACR, serum cholesterol, triglyceride, high-density lipoprotein cholesterol, low-density lipoprotein cholesterol, phosphorus, intact parathyroid hormone (iPTH), and fasting glucose levels. In addition, patients with higher baseline APAR levels (top binary) were more likely to have lower eGFR, hemoglobin, and serum calcium levels and lower frequencies of renin-angiotensin-aldosterone system inhibitor (RAASi) use (Table 1).

**Table 1:** Baseline characteristics by alkaline phosphatase-to-albumin ratio binary.

**Variable**	**Total (*n* = 2,081)**	**APAR, binary**		***P-*value**
		**Bottom binary (APAR** ≤ **1.737) (*****n*** = **1,041)**	**Top binary (APAR** > **1.737) (*****n*** = **1,040)**	
Age (years)	50.36 ± 14.13	48.41 ± 14.06	52.30 ± 13.96	< 0.001
**Sex (%)**				0.004
Male	1,213 (58.29%)	574 (55.14%)	639 (61.44%)	
Female	868 (41.71%)	467 (44.8%)	401 (38.56%)	
BMI (kg/m^2^)	24.58 ± 3.62	24.44 ± 3.60	24.72 ± 3.64	0.134
Diabetes (%)	436 (20.95%)	177 (17.00%)	259 (24.90%)	< 0.001
Hypertension (%)	1,488 (71.50%)	728 (69.93%)	760 (73.08%)	0.017
History of cardiovascular disease (%)	207 (9.95%)	91 (8.74%)	116 (11.15%)	0.078
Tobacco use (yes) (%)	609 (29.26%)	282 (27.09%)	327 (31.44%)	0.083
Serum creatinine (μmol/L)	145.80 (101.00, 205.00)	136.00 (98.00, 189.05)	153.00 (106.15, 222.68)	< 0.001
eGFR (ml/min/1.73 m^2^)	50.26 ± 30.21	52.96 ± 30.22	47.56 ± 29.97	< 0.001
ACR (mg/g)	388.69 (101.95, 951.70)	335.52 (86.95, 799.00)	472.82 (120.90, 1,172.46)	< 0.001
Hemoglobin (g/L)	126.65 ± 23.07	127.77 ± 21.74	125.48 ± 24.33	0.035
Neutrophil-to-lymphocyte ratio	2.28 (2.05, 2.51)	2.28 (2.01, 2.52)	2.28 (2.09, 2.49)	0.607
Serum cholesterol (mmol/L)	4.71 (3.96, 5.64)	4.62 (3.91, 5.49)	4.85 (4.01, 5.86)	0.001
Serum triglyceride (mmol/L)	1.76 (1.22, 2.47)	1.66 (1.16, 2.38)	1.84 (1.29, 2.58)	< 0.001
HDL-C (mmol/L)	1.08 (0.90, 1.31)	1.10 (0.91, 1.32)	1.06 (0.89, 1.29)	0.049
LDL-C (mmol/L)	2.64 (2.09, 3.32)	2.58 (2.02, 3.16)	2.73 (2.16, 3.45)	< 0.001
Serum calcium (mmol/L)	2.25 (2.14, 2.35)	2.27 (2.18, 2.36)	2.23 (2.10, 2.33)	< 0.001
Serum phosphorus (mmol/L)	1.19 (1.05, 1.33)	1.17 (1.04, 1.31)	1.20 (1.05, 1.35)	0.012
iPTH (pg/ml)	49.25 (31.41, 80.30)	45.39 (29.26, 69.13)	54.27 (34.18, 91.13)	< 0.001
Fasting glucose (mmol/L)	5.03 (4.53, 5.72)	5.00 (4.52, 5.59)	5.07 (4.54, 5.90)	0.029
**Staging of CKD (%)**				< 0.001
Stage 1	286 (13.74%)	146 (14.02%)	140 (13.46%)	
Stage 2	351 (16.87%)	217 (20.85%)	134 (12.88%)	
Stage 3a	326 (15.67%)	175 (16.81%)	151 (14.52%)	
Stage 3b	487 (23.40%)	234 (22.48%)	253 (24.33%)	
Stage 4	631 (30.32%)	269 (25.84%)	362 (34.81%)	
**Medication**				
Statin use (%)	459 (22.06%)	243 (23.34%)	216 (20.77%)	0.173
RAASi use (%)	1,098 (52.76%)	627 (60.23%)	471 (45.29%)	< 0.001
Active vitamin D use (%)	380 (18.26%)	191 (18.35%)	189 (18.17%)	0.918
**CKD etiologies (%)**				< 0.001
Primary glomerulonephritis	1,091 (52.43%)	592 (56.87%)	499 (47.98%)	
Diabetic kidney disease	246 (11.82%)	77 (7.40%)	169 (16.25%)	
Others	479 (23.02%)	241 (23.15%)	238 (22.88%)	

Continuous variables were reported by mean ± standard deviation or median (Quartile 1 and Quartile 3); categorical variables by count (percentage).

Missing value: BMI (531), diabetes (63), hypertension (210), tobacco use (288), ACR (221), hemoglobin (289), neutrophil-to-lymphocyte ratio (786), serum cholesterol (118), serum triglyceride (126), HDL-C (214), LDL-C (206), serum calcium (49), serum phosphorus (66), iPTH (246), fasting glucose (70), and CKD etiologies (265).

BMI, body mass index; eGFR, estimated glomerular filtration rate; ACR, albumin-to-creatinine ratio; HDL-C, high-density lipoprotein cholesterol; LDL-C, low-density lipoprotein cholesterol; iPTH, intact parathyroid hormone; RAASi, renin-angiotensin-aldosterone system inhibitor; CKD, chronic kidney disease.

### 3.3. Incidence rates of end-point events by APAR

In this study, 103 (4.95%) participants were lost to follow-up. The median follow-up time for ESKD, MACCEs, and all-cause death was 4.96 years (interquartile range: 3.82–5.92 years), 5.19 years (4.08–5.97 years), and 5.31 years (4.23–5.97 years), respectively. Of the 304 ESKD events, 211 participants entered maintenance hemodialysis, 80 entered peritoneal dialysis, and 13 were kidney transplant recipients. The 141 MACCEs consisted of 14 cases of acute myocardial infarction, 22 cases of unstable angina pectoris, 47 cases of congestive heart failure requiring hospitalization, 46 cerebrovascular events, 11 cases of severe arrhythmia, and 1 case of peripheral arterial diseases. In total, 73 patients died, including 19 deaths due to CVD (Supplementary Table 2). The overall incidence rates of ESKD, MACCEs, and all-cause death were 3.19, 1.40, and 0.70 per 100 person-years, respectively (Table 2). Among APAR quartile groups, the incidences of ESKD, MACCEs, and all-cause death were highest in the Quartile 4 group, and *P*-values were all <0.05 by log-rank test (Table 2).

**Table 2:** Incidence of end-point events by alkaline phosphatase-to-albumin ratio quartile.

**APAR (n)**	**ESKD (RRT)**			**MACCE**			**All-cause death**		
	**Events** ***n*** **(%)**	**Incidence rates (/100 person-years)**	^*^ ***P-*** **value**	**Events** ***n*** **(%)**	**Incidence rates (/100 person-years)**	^*^ ***P-*** **value**	**Events** ***n*****(%)**	**Incidence rates (/100 person-years)**	^*^ ***P-*** **value**
Quartile 1 (*n* = 521)	55 (10.56)	2.14	< 0.001	26 (4.99)	0.98	0.002	12 (2.30)	0.44	< 0.001
Quartile 2 (*n* = 520)	65 (12.5)	2.64		28 (5.38)	1.09		11 (2.12)	0.42	
Quartile 3 (*n* = 520)	87 (16.73)	3.69		37 (7.12)	1.48		17 (3.27)	0.65	
Quartile 4 (*n* = 520)	97 (18.65)	4.54		50 (9.62)	2.16		33 (6.35)	1.36	
Total (*n* = 2,081)	304 (14.61)	3.19		141 (6.78)	1.40		73 (3.51)	0.70	

* *P*-value was tested by log-rank test.

APAR, alkaline phosphatase-to-albumin ratio; ESKD, end-stage kidney disease; RRT, renal replacement therapy; MACCE, major adverse cardiovascular and cerebrovascular events.

### 3.4. Association with end-point outcomes

#### 3.4.1. Association of APAR (continuous variable) with end-point outcomes

**Figure 2:**
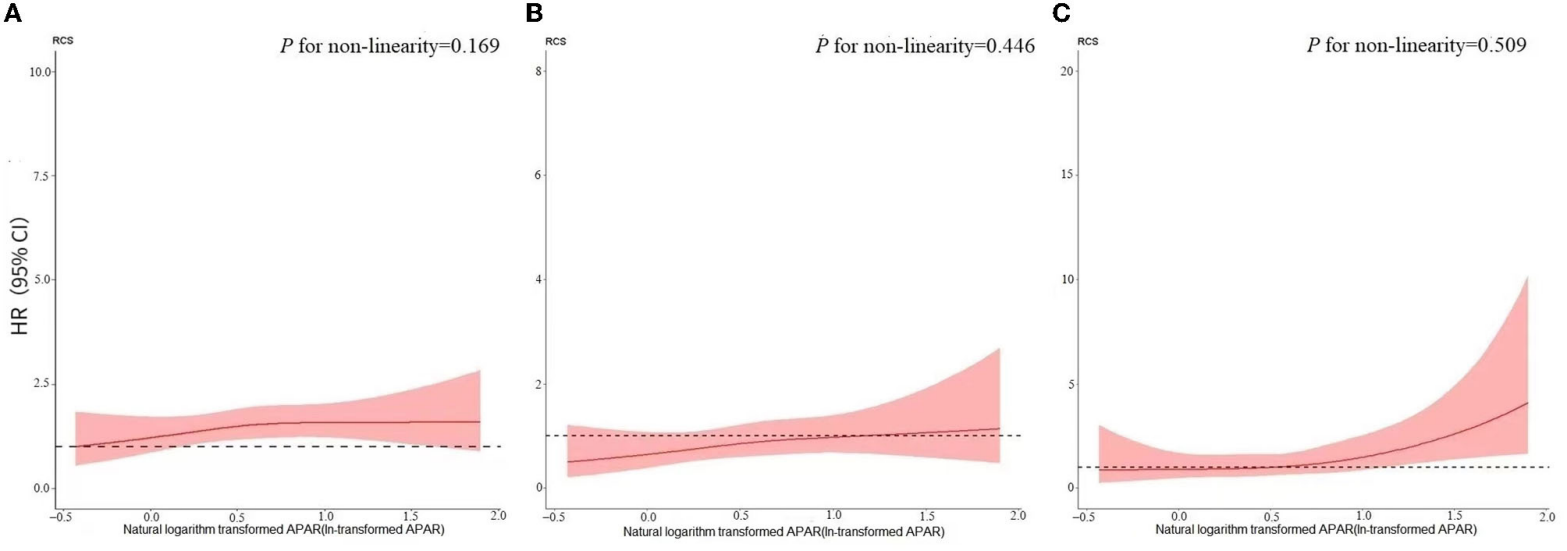
Restricted cubic spline for the relationship between APAR and **(A)** ESKD:RRT; **(B)** MACCE, and **(C)** all-cause death. Models adjusted for age, sex, BMI, tobacco use, hypertension, diabetes, CVD history, CKD etiologies, RAASi use, ln-transformed LDL-C, ln-transformed ACR, hemoglobin, and eGFR. **(A)** *P* for non-linearity = 0.169; **(B)** *P* for non-linearity = 0.446; **(C)** *P* for non-linearity = 0.509. APAR, alkaline phosphatase-to-albumin ratio; ESKD, end-stage kidney disease; RRT, renal replacement therapy; MACCEs, major adverse cardiovascular and cerebrovascular events; BMI, body mass index; CVD, cardiovascular disease; RAASi, renin-angiotensin-aldosterone system inhibitor; ln-transformed, natural logarithm transformed; LDL-C, low-density lipoprotein cholesterol; ACR, albumin-to-creatinine ratio; eGFR, estimated glomerular filtration rate.

Monotonic relationships between ln-transformed APAR levels and end-point outcomes were confirmed by the RCS of the non-linear test (Figure 2; all *P* for non-linearity > 0.05). In the primary model (adjusted model 3), the increasing APAR levels were not found to be associated with a higher risk of new occurrences of ESKD (SHR, 1.061; 95% CI, 0.938–1.201) and MACCEs (SHR, 1.118; 95% CI, 0.956–1.308), as shown in Table 3. For the end-point outcome of all-cause death, the association of APAR values with death was consistent both in the unadjusted and adjusted models. In the primary model (adjusted model 3), increasing the APAR value [each 1-standard deviation (SD) increase in ln-transformed measurement] was associated with a 33.5% higher hazard of death (HR, 1.335; 95% CI, 1.068–1.670). The SD of ln-transformed APAR was 0.387.

**Table 3:** Unadjusted and adjusted hazard of **(A)** end-stage kidney disease: renal replacement therapy; **(B)** major adverse cardiovascular and cerebral events; and **(C)** all-cause death, by alkaline phosphatase-to-albumin ratio levels (continuous/categorical variable).

**(A) End-stage kidney disease: renal replacement therapy**										
**APAR (continuous)**	**Unadjusted model**		**Adjusted model 1** ^a^		**Adjusted model 2** ^b^		**Adjusted model 3** ^c^		**Exploratory model** ^c^	
	**SHR (95% CI)**	***P-*** **value**	**SHR (95% CI)**	***P-*** **value**	**SHR (95% CI)**	***P-*** **value**	**SHR (95% CI)**	***P-*** **value**	**SHR (95% CI)**	***P-*** **value**
	1.220 (1.100–1.360)	< 0.001	1.231 (1.109–1.365)	< 0.001	1.078 (0.960–1.211)	0.210	1.061 (0.938–1.201)	0.340	1.061 (0.939–1.199)	0.340
**APAR (quartile)**	**Unadjusted model**		**Adjusted model 1** ^a^		**Adjusted model 2** ^b^		**Adjusted model 3** ^c^		**exploratory model** ^c^	
	**SHR (95% CI)**	***P*** **for trend**	**SHR (95% CI)**	***P*** **for trend**	**SHR (95% CI)**	***P*** **for trend**	**SHR (95% CI)**	***P*** **for trend**	**SHR (95% CI)**	***P*** **for trend**
Quartile 1 (*n* = 521)	1.000 (reference)	< 0.001	1.000 (reference)	< 0.001	1.000 (reference)	0.07	1.000 (reference)	0.15	1.000 (reference)	0.15
Quartile 2 (*n* = 520)	1.230 (0.861–1.760)		1.241 (0.865–1.779)		1.081 (0.754–1.549)		1.030 (0.722–1.471)		1.038 (0.727–1.481)	
Quartile 3 (*n* = 520)	1.710 (1.223–2.390)		1.748 (0.238–2.469)		1.443 (1.021–2.040)		1.378 (0.983–1.931)		1.382 (0.987–1.936)	
Quartile 4 (*n* = 520)	2.070 (1.484–2.870)		2.137 (1.521–3.002)		1.367 (0.947–1.973)		1.255 (0.878–1.793)		1.259 (0.882–1.795)	
**(B) Major adverse cardiovascular and cerebral events**										
**APAR (continuous)**	**Unadjusted model**		**Adjusted model 1**		**Adjusted model 2** ^d^		**Adjusted model 3** ^d^		**Exploratory model** ^d^	
	**SHR (95% CI)**	***P-*** **value**	**SHR (95% CI)**	***P-*** **value**	**SHR (95% CI)**	***P-*** **value**	**SHR (95% CI)**	***P-*** **value**	**SHR (95% CI)**	***P-*** **value**
	1.230 (1.080–1.410)	0.003	1.180 (1.015–1.370)	0.031	1.114 (0.956–1.298)	0.170	1.118 (0.956–1.308)	0.160	1.120 (0.959–1.307)	0.150
**APAR (quartile)**	**Unadjusted model**		**Adjusted model 1**		**Adjusted model 2** ^d^		**Adjusted model 3** ^d^		**Exploratory model** ^d^	
	**SHR (95% CI)**	***P*** **for trend**	**SHR (95% CI)**	***P*** **for trend**	**SHR (95% CI)**	***P*** **for trend**	**SHR (95% CI)**	***P*** **for trend**	**SHR (95% CI)**	***P*** **for trend**
Quartile 1 (*n* = 521)	1.000 (reference)	< 0.001	1.000 (reference)	0.006	1.000 (reference)	0.052	1.000 (reference)	0.056	1.000 (reference)	0.055
Quartile 2 (*n* = 520)	1.110 (0.649–1.890)		1.020 (0.595–1.750)		0.999 (0.587–1.700)		1.002 (0.589–1.707)		1.011 (0.593–1.724)	
Quartile 3 (*n* = 520)	1.500 (0.907–2.470)		1.290 (0.776–2.140)		1.207 (0.728–2.002)		1.188 (0.715–1.971)		1.199 (0.722–1.993)	
Quartile 4 (*n* = 520)	2.170 (1.350–3.490)		1.770 (1.088–2.880)		1.503 (0.927–2.437)		1.493 (0.921–2.421)		1.499 (0.927–2.427)	
**(C) All-cause death**										
**APAR (continuous)**	**Unadjusted model**		**Adjusted model 1** ^e^		**Adjusted model 2** ^e^		**Adjusted model 3** ^e^		**Exploratory model** ^e^	
	**HR (95% CI)**	***P-*** **value**	**HR (95% CI)**	***P-*** **value**	**HR (95% CI)**	***P-*** **value**	**HR (95% CI)**	***P-*** **value**	**HR (95% CI)**	***P-*** **value**
	1.558 (1.291–1.881)	< 0.001	1.506 (1.229–1.845)	< 0.001	1.335 (1.068–1.669)	0.011	1.335 (1.068–1.670)	0.011	1.332 (1.067–1.664)	0.011
Quartile 1 (*n* = 521)	1.000 (reference)	< 0.001	1.000 (reference)	< 0.001	1.000 (reference)	0.025	1.000 (reference)	0.025	1.000 (reference)	0.026
Quartile 2 (*n* = 520)	0.950 (0.419–2.154)		0.836 (0.369–1.895)		0.757 (0.332–1.724)		0.756 (0.332–1.723)		0.770 (0.338–1.757)	
Quartile 3 (*n* = 520)	1.522 (0.727–3.188)		1.214 (0.579–2.548)		1.091 (0.517–2.303)		1.092 (0.518–2.305)		1.122 (0.530–2.375)	
Quartile 4 (*n* = 520)	3.293 (1.699–6.384)		2.509 (1.291–4.879)		1.728 (0.867–3.446)		1.727 (0.866–3.445)		1.738 (0.872–3.463)	

Subhazard ratios are from competing risk models, allowing for death as a competing factor. Hazard ratios are from the standard Cox proportional regression model. SHR/HR expressed per 1-standard deviation greater ln-transformed APAR level (continuous variable). Adjusted model 1 adjusted for age and sex. Adjusted model 2 adjusted for age, sex, BMI, tobacco use, hypertension, diabetes, CVD, CKD etiologies, ln-transformed LDL-C, ln-transformed ACR, RAASi use, and hemoglobin. Adjusted model 3 adjusted for age, sex, BMI, tobacco use, hypertension, diabetes, CVD, CKD etiologies, ln-transformed LDL-C, ln-transformed ACR, RAASi use, hemoglobin, and eGFR. The exploratory model further adjusted for ln-transformed NLR on the basis of model 3.

a Age is included as a time-varying covariate.

b Age, RAASi use, and ln-transformed ACR are included as time-varying covariates.

c RAASi use and ln-transformed ACR are included as time-varying covariates.

d Hemoglobin is included as a time-varying covariate.

e Sex is included as a time-varying covariate.

CKD, chronic kidney disease; APAR, alkaline phosphatase-to-albumin ratio; SHR, subhazard ratio; HR, hazard ratio; CI, confidence interval; ln-transformed, natural logarithm transformed; BMI, body mass index; CVD, cardiovascular disease; RAASi, renin-angiotensin-aldosterone system inhibitor; LDL-C, low-density lipoprotein cholesterol; ACR, albumin-to-creatinine ratio; eGFR, estimated glomerular filtration rate; NLR, neutrophil-to-lymphocyte ratio.

#### 3.4.2. Association of APAR (categorical variable) with end-point

The continuous variable, APAR, was categorized into quartiles. Category boundaries of Quartile 1 were ≤1.379, category boundaries of Quartile 2 ranged from 1.379 to 1.737, those of Quartile 3 were >1.737 and ≤2.205, and those of Quartile 4 ranged from 2.205 to 12.053. As reported in Table 3, a stepwise increase in the risk of ESRD and MACCEs was not observed for increasing quartiles of APAR in the primary model (*P*-values for trend > 0.05). Compared with APAR in Quartile 1, APAR in Quartile 4 did not appear to be statistically associated with an increased hazard of ESRD (HR, 1.255; 95% CI, 0.878–1.793) and MACCEs (HR, 1.493; 95% CI, 0.921–2.421) in the primary model (Table 3). As for all-cause death, APAR in Quartile 4 was not found to have an increased hazard of death (HR, 1.727; 95% CI, 0.866–3.445) compared to APAR in Quartile 1 in the primary model (Table 3). Interestingly, we found evidence for a linear association between increasing APAR quartiles and all-cause death risk elevation both in unadjusted and multivariable models (*P*-values for trend were all statistically significant).

### 3.5. Subgroup analyses

There was evidence of effect modification on the relationship between APAR levels and ESRD by baseline eGFR (*P*-interaction < 0.001). We used the eGFR (60 ml/min/1.73 m^2^) as the cutoff value. Every 1-SD greater ln-transformed APAR value was associated with greater risk for ESRD among participants with eGFR ≥ 60 ml/min/1.73 m^2^: SHRs of 1.654 (95% CI, 1.243–2.202). Nevertheless, APAR levels were not associated with the risk of ESRD in participants with eGFR < 60 ml/min/1.73 m^2^. Table 4 presents the effect estimates for subgroups.

**Table 4:** Adjusted hazard of end-point events by natural logarithm transformed alkaline phosphatase-to-albumin ratio levels stratified by baseline age/sex/eGFR/CVD/CKD etiologies (subgroup analyses).

**End-point events**	**Subgroup**	**SHR / HR (95% CI), *P*-value**	***P-*interaction**
**End-stage kidney disease**		**SHR (95% CI)**, ***P*****-value**	
Age			0.113
	Age ≥ 51 years (median)	0.979 (0.827–1.157)^a^; ^*^P = 0.800	
	Age < 51 years (median)	1.149 (0.967–1.365); ^*^P = 0.120	
Sex			0.341
	Male	1.040 (0.873–1.230)^b^; ^#^*P* = 0.690	
	Female	1.174 (0.984–1.402)^c^; ^#^*P* = 0.075	
eGFR			< 0.001
	eGFR ≥ 60 ml/min/1.73 m^2^	1.880 (1.260–2.810)^d^; ^*^P = 0.002	
	eGFR < 60 ml/min/1.73 m^2^	1.013 (0.895–1.147)^a^; ^*^P = 0.830	
History of CVD			0.095
	Yes	0.864 (0.691–1.080)^e^; ^■^*P* = 0.200	
	No	1.127 (0.989–1.284)^f^; ^■^*P* = 0.074	
CKD etiologies			0.462
	Diabetic kidney disease	0.930 (0.750–1.150)^g^; ^§^*P* = 0.510	
	Non-diabetic kidney disease	1.109 (0.971–1.267)^h^; ^§^*P* = 0.130	
**MACCE**	**Subgroup**	**SHR (95% CI)**, ***P*****-value**	***P-*** **interaction**
Age			0.398
	Age ≥ 51 years (median)	1.109 (0.900–1.370); ^*^P = 0.330	
	Age < 51 years (median)	1.050 (0.818–1.350)^i^; ^*^P = 0.700	
Sex			0.310
	Male	1.134 (0.919–1.400); ^#^*P* = 0.240	
	Female	1.144 (0.875–1.495)^j^; ^#^*P* = 0.330	
eGFR			0.569
	eGFR ≥ 60 ml/min/1.73 m^2^	1.330 (0.825–2.150); ^*^P = 0.240	
	eGFR < 60 ml/min/1.73 m^2^	1.098 (0.928–1.300); ^*^P = 0.280	
History of CVD			0.057
	Yes	0.983 (0.728–1.330)^f^; ^■^*P* = 0.910	
	No	1.196 (0.999–1.431); ^■^*P* = 0.051	
CKD etiologies			0.816
	Diabetic kidney disease	1.145 (0.829–1.580); ^§^*P* = 0.410	
	Non-diabetic kidney disease	1.103 (0.906–1.340)^f^; ^§^*P* = 0.330	
**All-cause death**	**Subgroup**	**HR (95% CI)**, ***P-*****value**	***P*** **-interaction**
Age			0.146
	Age ≥ 51 years (median)	1.330 (0.984–1.790)^k^; ^*^P = 0.063	
	Age < 51 years (median)	1.229 (0.777–1.943); ^*^P = 0.378	
Sex			0.164
	Male	1.513 (1.127–2.031); ^#^*P* = 0.006	
	Female	1.096 (0.777–1.545); ^#^*P* = 0.601	
eGFR			0.007
	eGFR ≥ 60 ml/min/1.73 m^2^	1.661 (1.049–2.629); ^*^P = 0.030	
	eGFR < 60 ml/min/1.73 m^2^	1.216 (0.946–1.562)^k^; ^*^P = 0.126	
History of CVD			0.804
	Yes	1.679 (0.749–3.764)^l^; ^■^*P* = 0.209	
	No	1.079 (0.999–1.625); ^■^*P* = 0.052	
CKD etiologies			0.008
	Diabetic kidney disease	2.093 (1.292–3.389); ^§^*P* = 0.003	
	Non-diabetic kidney disease	1.186 (0.913–1.542)^l^; ^§^*P* = 0.201	

Subhazard ratios were from competing risk models, allowing for death as competing. Hazard ratios were from the standard Cox proportional regression model. SHR/HR expressed per 1-standard deviation greater ln-transformed APAR levels.

* Models adjusted for age, sex, BMI, tobacco use, hypertension, diabetes, CVD history, CKD etiologies, ln-transformed LDL-C, ln-transformed ACR, RAASi use, hemoglobin, and eGFR.

# Models adjusted for age, BMI, tobacco use, hypertension, diabetes, CVD history, CKD etiologies, ln-transformed LDL-C, ln-transformed ACR, RAASi use, hemoglobin, and eGFR.

■ Models adjusted for age, sex, BMI, tobacco use, hypertension, ln-transformed LDL-C, ln-transformed ACR, RAASi use, hemoglobin, and eGFR.

§ Models adjusted for age, sex, BMI, tobacco use, hypertension, CVD history, ln-transformed LDL-C, ln-transformed ACR, RAASi use, hemoglobin, and eGFR.

a RAASi use, ln-transformed ACR, and eGFR are included as time-varying covariates.

b CKD etiologies and RAASi use are included as time-varying covariates.

c Age and RAASi use are included as time-varying covariates.

d CKD etiologies are included as a time-varying covariate.

e Ln-transformed ACR and ln-transformed LDL-C are included as time-varying covariates.

f eGFR is included as a time-varying covariate.

g Sex and CVD are included as time-varying covariates.

h Age, eGFR, and RAASi use are included as time-varying covariates.

i Hemoglobin and eGFR are included as time-varying covariates.

j Hemoglobin is included as a time-varying covariate.

k Sex and hemoglobin are included as time-varying covariates.

l Sex is included as a time-varying covariate.

eGFR, estimated glomerular filtration rate; CVD, cardiovascular disease; CKD, chronic kidney disease; SHR, subhazard ratio; HR, hazard ratio; CI, confidence interval; MACCE, major adverse cardiovascular and cerebral events; BMI, body mass index; LDL-C, low-density lipoprotein cholesterol; ACR, albumin-to-creatinine ratio; RAASi, renin-angiotensin-aldosterone system inhibitor; ln-transformed, natural logarithm transformed.

In addition, we found evidence for interaction among eGFR, CKD etiologies, and APAR levels for the risk of all-cause death (Table 4). Each 1-SD greater ln-transformed APAR value was associated with a 66.1% higher risk of all-cause death (95% CI, 1.049–2.629) among participants with eGFR ≥ 60 ml/min/1.73 m^2^. However, the association was not present in patients with eGFR < 60 ml/min/1.73 m^2^. Moreover, we also found that the risk of all-cause death was elevated by 2.093-fold (95% CI, 1.292–3.389) for every 1-SD increase in ln-transformed APAR value among patients with DKD, but not in non-DKD patients. Subgroup analyses stratified by sex are listed in Table 4. The increasing APAR levels were found to be associated with a higher risk of death (HR, 1.513; 95% CI, 1.127–2.031) in male participants, whereas the association did not exist in female participants.

### 3.6. Sensitivity analysis

Given that APAR partly reflects the inflammatory state, we further adjusted the indicator of inflammatory, neutrophil-to-lymphocyte ratio (NLR), based on the primary model as an exploratory model to explore the association between APAR levels and end-point events. The general association between APAR levels and all-cause death persisted in the exploratory model regardless of whether APAR was a continuous variable or a categorical variable. We found that each 1-SD greater ln-transformed APAR value was associated with a 33.2% higher hazard of death (95% CI, 1.067–1.664). Compared with APAR in the bottom quartile (Quartile 1), a stepwise increase in the risk of death was observed for increasing quartiles of APAR (*P*-values for trend < 0.05). However, the associations between higher APAR levels and increased ESRD and MACCE risks were not statistically significant in the exploratory model (Table 4).

## 4. Discussion

In this analysis of 2,180 patients with CKD stages 1–4 from the C-STRIDE prospective cohort study, the overall incidence rate of ESKD, MACCEs, and all-cause death were 3.19, 1.40, and 0.7 per 100 person-years, respectively. In addition, the incidence of all three outcomes increased with increasing APAR levels. A Chronic Kidney Disease Japan Cohort (CKD-JAC) study of 2,966 Japanese patients with CKD (eGFR 10–59 ml/min/1.73 m^2^) over 4 years showed a 0.72 per 100 person-years incidence of all-cause death (24). This result is similar to our study result. In addition, the Chronic Renal Insufficiency Cohort (CRIC) study is a prospective cohort study of adults with CKD conducted at seven US clinical centers. CRIC enrolled 3,939 adults with mild-to-moderate CKD (eGFR 20–70 ml/min/1.73 m^2^). Among the 3,739 CRIC participants, the crude all-cause mortality rate was 3.16 per 100 person-years during 6.8 years of median follow-up (25). The higher incidence of death in the CRIC study may be related to the underlying etiologies of chronic renal insufficiency. In particular, the proportion of patients with DM in the CRIC study was 48.4%, which was significantly higher than the 11.8% in the C-STRIDE study. The presence of DM in patients with CKD greatly affects the prognosis. The risk of death was obviously higher in patients who had diabetic nephropathy than in patients who did not. A nationwide study in Sweden showed that, overall, among patients with CKD stages 3b−5 (7,388 patients with stage 3b, 18,282 with stage 4, and 9,410 with stage 5), the incidence rate of deaths was 10.1 per 100 person-years (26). The lower incidence of death in the C-STRIDE study may be related to the inclusion of patients with early-stage CKD (stages 1–4).

We analyzed the association between APAR levels and the prognosis of CKD in this study. ALP is a membrane-bound metalloenzyme that consists of a group of isoenzymes. Each isoenzyme is a glycoprotein encoded by different gene loci, encoded for by at least four different gene loci: tissue-nonspecific, intestinal, placental, and germ-cell ALP (5). Tissue-non-specific ALP is highly expressed in bone and liver tissues, accounting for ~95% of total serum ALP activity. Disordered serum ALP is involved in chronic inflammation, MBD disorder, and vascular calcification of CKD (27). We measured serum total ALP and did not subdivide the isoenzymes of ALP. Studies have indicated that higher serum total ALP levels were associated with increased mortality in hemodialysis or peritoneal dialysis patients (28–30). For patients with early-stage CKD, there are fewer relevant findings. Serum ALB is synthesized by hepatocytes that manifest the condition of nutrition and antioxidant effect. Decreased albumin levels are strongly associated with chronic inflammation and vascular calcification (7, 31). Studies have reported that decreased ALB has been confirmed to be an independent predictor of mortality from certain diseases (32, 33). ALP associated with ALB (APAR) indicator is a new marker logically derived. APAR as a predictor has been applied to several cancers and ACS (11–14).

The current study suggested that the increasing APAR levels were not found to be associated with a higher risk of a new occurrence of ESKD. When APAR was a continuous variable, higher APAR levels were associated with increased risk of ESKD events only in the unadjusted model and model 1 adjusted for age and sex. However, the association was not statistically significant after further adjustment for traditional risk factors of cardiovascular events. Then, after further adjustment for eGFR, no correlation was found. When APAR was used as a categorical variable, the results of the trend test were the same as those of a continuous variable, and no correlation was found. Considering the inflammatory effect of the APAR indicator, we attempted a sensitivity analysis to verify the robustness of the results. Combined with previous research results, NLR (an indicator of non-specific inflammation) may independently predict the risk of ESKD in patients with stage 4 CKD (34). We added NLR as a covariable based on the primary model. In general, the main findings in the primary model and exploratory model were consistent. More importantly, there was evidence for effect modification according to baseline eGFR. Subgroup analysis demonstrated that every 1-SD greater ln-transformed APAR value was associated with a greater risk for ESKD among participants with eGFR ≥ 60 ml/min/1.73 m^2^. Nevertheless, the association was not found in patients with eGFR < 60 ml/min/1.73 m^2^. This was a very interesting discovery. It is possible that patients with eGFR < 60 ml/min/1.73 m^2^ were more likely to be complicated with many comorbidities and complications. After adjusting for confounding factors such as comorbidities, the association between APAR and ESKD may be greatly weakened. In contrast, in patients with eGFR ≥ 60 ml/min/1.73 m^2^, the association may be more significant and independent.

Several studies suggested that ALP has a predictive value for MACCEs in the general population and ESKD population (35, 36). In a previous study with a median follow-up of 125 months, hypoproteinemia was not an independent risk factor for cardiovascular mortality in CKD stages 3 and 4 (37). In the C-STRIDE study, we did not observe any significant associations between APAR and the risk of non-fatal MACCEs in stages 1–4 CKD patients in general and CKD patients subgrouped according to age, sex, CVD history, and eGFR. The correlation between APAR levels and MACCEs among patients with CKD stages 1–4 was found only in the unadjusted model and adjusted model 1 (adjustment for demographic factor). However, after further adjustment for traditional cardiovascular risk factors, renal function, and inflammation indexes, the association was not statistically significant in this study. The spectrum for the etiology of CKD in China differs from that in Western countries. Despite the increasing prevalence of diabetes from the 1980s onwards leading to more patients being affected by DKD (2), glomerulonephritis may still account for a considerable proportion of patients with CKD in China (38–40). In general, patients with DKD were often older, had more comorbidities, and were more prone to CVD events than those with glomerulonephritis. In our study, a higher proportion of subjects with nephritis were included, which may have had some impact on the results. Additionally, MACCE was a composite outcome that mainly included non-fatal CVD events in this study. For the outcome of cardiovascular death, we did not analyze the correlation between APAR levels and its occurrence due to the small number of cases. Therefore, the relationship between them needs to be further confirmed in the future.

This study preliminarily demonstrated the correlation between APAR levels and the risk of death among patients with stages 1–4 CKD. The results showed that higher APAR levels were associated with an increased risk of all-cause death among stages 1–4 CKD, independent of demographic characteristics, traditional risk factors of cardiovascular events, renal function indexes, and non-specific inflammatory indicators. Specifically, when APAR was analyzed as a categorical variable, although there was no significant difference in the risk of death in Quartiles 2–4 compared with Quartile 1, the results of the trend test were statistically significant. This may be due to the small sample of deaths and relatively short follow-up duration. Overall, the main results of APAR as a continuous variable and as a categorical variable were consistent. Of note, for the all-cause outcome, there was evidence for effect modification according to baseline eGFR and CKD etiologies. In subgroup analyses, we found that higher APAR levels were associated with an increased risk of death in male patients, in DKD patients, and in patients with eGFR > 60 ml/min/1.73 m^2^. However, there was no association between higher APAR levels and an increased risk of death in CKD patients who were women, non-DKD, or had lower eGFR levels. The results from patients with subgroups should be applied with caution and should be validated by future studies with larger samples and longer follow-up times. In general, APAR can be used in risk assessment for all-cause death among patients with CKD; this application may be appealing considering APAR is a routine test.

There were several limitations in this study. Above all, the median follow-up time for the participants was ~5 years. In view of the much longer time needed to reach an endpoint for CKD, follow-up time may result in a limited number of endpoints, which may restrict the statistical power of the study. Second, baseline data of APAR were missing for some patients across the whole cohort. Even if we compared the two data sets and adjusted for relevant confounding factors, it was still a limitation. Third, all data in this study were collected from China and all subjects comprised of Asian people. A large-scale and multicenter prospective study should be conducted worldwide to verify our results and eliminate the selective bias. Fourth, this study only referred to the APAR values at the baseline and lacked repeated measures of APAR. At last, although we adjusted for apparent confounders, potential residual confounders remained.

In conclusion, our results suggested that higher APAR levels in patients with CKD stages 1–4 appeared to be associated with an increased risk for all-cause death. Moreover, subgroup analyses demonstrated that an elevated APAR value was related to ESKD among patients with CKD with higher eGFR levels, but no relationship was observed in those with lower eGFR. Since the test is inexpensive and available, it can be used in risk assessment for all-cause death among patients with CKD stages 1–4. These findings should be further validated in multicenter, large-sample prospective studies with longer follow-up.

## Data availability statement

The data presented in this study are included in the article and Supplementary material, further inquiries can be directed to the corresponding author. Requests to access these datasets should be directed to X-QW (wangxiaoqin@hbhtcm.com).

## Ethics statement

The studies involving humans were approved by Ethics Committee of Peking University First Hospital. The studies were conducted in accordance with the local legislation and institutional requirements. The participants provided their written informed consent to participate in this study.

## Author contributions

The C-STRIDE study was completed by 39 clinical centers in China. M-HZ and L-XZ are the main initiators and designers of the C-STRIDE study. X-QW is the head of the subcentre of Hubei. In this study, statistical analyses were conducted by J-WW and XX. JY, HC, X-RZ, L-ML, D-FD, W-CX, and YZ were primarily responsible for subject recruitment and data entry. XX and J-XL were responsible for writing this article. M-HZ, L-XZ, and X-QW were responsible for the critical revision of the manuscript. All authors have contributed to the manuscript and approved the submitted version.
