# Supplementary material for: Association between alkaline phosphatase/albumin ratio and the prognosis in patients with chronic kidney disease stages 1–4: results from a C-STRIDE prospective cohort study
Source: Front Med (Lausanne). 2023 Sep 20;10:1215318. doi: 10.3389/fmed.2023.1215318 (PMC10548241; doi:10.3389/fmed.2023.1215318)
Supplement: Supplementary file 1 [file Table_1.DOCX]

Supplementary Material

Association between alkaline phosphatase/albumin ratio and the prognosis in patients with chronic kidney disease stages 1-4: Results from the C-STRIDE prospective cohort study

Xue Xue^†^, Jia-Xuan Li^†^, Jin-Wei Wang, La-Mei Lin, Hong Cheng, Dan-Fang Deng, Wen-Cheng Xu, Yu Zhao, Xin-Rong Zou, Jun Yuan, Lu-Xia Zhang, Ming-Hui Zhao, and Xiao-Qin Wang*

*** Correspondence:** Xiao-Qin Wang: wangxiaoqin@hbhtcm.com

# Supplementary Table 1 Baseline characteristics of patients with chronic kidney disease included and not included in this study

| **Variable** | **The population not included in the analysis of this study**  **(n= 1619)** | **The population**  **included in the analysis of this study**  **(n= 2081)** | ***P***  **value** |
| --- | --- | --- | --- |
| Age (years) | 49.40±14.49 | 50.36±14.14 | 0.044 |
| Sex (%) |  |  | 0.945 |
| Male | 941(58.12%) | 1213(58.29%) |  |
| Female | 678(41.88%) | 868(41.71%) |  |
| BMI (kg/m^2^) | 24.53±3.63 | 24.58±3.62 | 0.711 |
| Diabetes (%) | 336(20.75%) | 436(20.95%) | 0.883 |
| Hypertension (%) | 1233(76.16%) | 1488(71.50%) | 0.756 |
| Cardiovascular disease (%) | 138(8.52%) | 207(9.95%) | 0.156 |
| Tobacco use (Yes) (%) | 509(31.44%) | 609(29.26%) | 0.638 |
| Serum creatinine (µmol/L) | 147.00(104.50,214.00) | 145.80(101.00,205.00) | 0.130 |
| eGFR (ml/min/1.73 m^2^) | 49.30±30.34 | 50.26±30.21 | 0.335 |
| ACR (mg/g) | 351.67(72.86,878.03) | 388.69(101.95,951.70) | 0.005 |
| Hemoglobin (g/L) | 126.84±22.61 | 126.65±23.07 | 0.810 |
| Neutrophil to lymphocyte ratio | 2.33(1.69,3.14) | 2.28(1.68,3.06) | 0.326 |
| Serum cholesterol (mmol/L) | 4.78(3.90,5.85) | 4.71(3.96,5.64) | 0.324 |
| Serum triglyceride (mmol/L) | 1.83(1.23,2.63) | 1.76(1.22,2.47) | 0.106 |
| HDL-C (mmol/L) | 1.06(0.89,1.31) | 1.08(0.90,1.31) | 0.300 |
| LDL-C (mmol/L) | 2.53(2.06,3.16) | 2.64(2.09,3.32) | 0.013 |
| Serum calcium (mmol/L) | 2.25(2.13,2.34) | 2.25(2.14,2.35) | 0.278 |
| Serum phosphorus (mmol/L) | 1.19(1.06,1.35) | 1.19(1.05,1.33) | 0.059 |
| iPTH (pg/ml) | 49.12(30.46,82.36) | 49.25(31.41,80.30) | 0.910 |
| Fasting glucose (mmol/L) | 4.98(4.40,5.74) | 5.03(4.53,5.72) | 0.052 |
| Medication |  |  |  |
| Statin use (%) | 329(20.32%) | 459(22.06%) | 0.216 |
| RAASi use (%) | 830(51.27%) | 1098(52.76%) | 0.384 |
| Active vitamin D use (%) | 274(16.92%) | 380(18.26%) | 0.290 |
| CKD etiologies (%) |  |  | 0.547 |
| Primary glomerulonephritis | 882(54.48%) | 1091(52.43%) |  |
| Diabetic kidney disease | 211(13.03%) | 246(11.82%) |  |
| Others | 363(22.42%) | 479(23.02%) |  |

**Note:** Continuous variables were reported by mean ± standard deviation or median (Quartile1, Quartile3); categorical variables by count (percentage).

**Missing value:** BMI(807), diabetes(493), hypertension(269), tobacco use(445), ACR(221), hemoglobin(395), neutrophil to lymphocyte ratio(786), serum cholesterol(118), serum triglyceride(126), HDL-C(214), LDL-C(206), serum calcium(49), serum phosphorus(66), iPTH(246), fasting glucose(70), CKD etiologies (428).

**Abbreviations:** BMI: body mass index; eGFR: estimated glomerular filtration rate; ACR: albumin -to-creatinine ratio; HDL-C: high-density lipoprotein cholesterol; LDL-C: low-density lipoprotein cholesterol; iPTH: intact parathyroid hormone; RAASi: renin-angiotensin-aldosterone system inhibitor; CKD: chronic kidney disease.

# Supplementary Table 2 Event counts by alkaline phosphatase-to-albumin ratio binary

| **Outcomes** | **Alkaline phosphatase-to-albumin ratio binary**  **(No. Events/No. Participants)** | |
| --- | --- | --- |
|  | **Bottom binary** | **Top binary** |
| **End-stage kidney disease** |  |  |
| Hemodialysis | 79 / 120 | 132 / 184 |
| Peritoneal dialysis | 35 / 120 | 45 / 184 |
| Renal transplantation | 6 / 120 | 7 / 184 |
| **Major adverse cardiovascular and cerebral events** |  |  |
| Acute myocardial infarction | 5 / 54 | 9 / 87 |
| Unstable angina pectoris | 10 / 54 | 12 / 87 |
| Congestive heart failure requiring hospitalization | 13 / 54 | 34 / 87 |
| Severe arrhythmia | 5 / 54 | 6 / 87 |
| Peripheral arterial disease | 1 / 54 | 0 / 87 |
| Cerebrovascular events | 20 / 54 | 26 / 87 |
| **All-cause death** |  |  |
| Cardiovascular disease | 6 / 23 | 13 / 50 |
| Others | 17 / 23 | 37/ 50 |
